# T Cells in Adipose Tissue: Critical Players in Immunometabolism

**DOI:** 10.3389/fimmu.2018.02509

**Published:** 2018-10-30

**Authors:** Qun Wang, Huaizhu Wu

**Affiliations:** ^1^Key Laboratory of Infection and Immunity of Shandong Province, Department of Immunology, School of Basic Medical Sciences, Shandong University, Jinan, China; ^2^Department of Medicine and Department of Pediatrics, Baylor College of Medicine, Houston, TX, United States

**Keywords:** T cell, adipose tissue, immunometabolism, obesity, insulin resistance

## Abstract

Adipose tissue performs immunoregulatory functions in addition to fat storage. Various T cells in different fat depots either help maintain metabolic homeostasis under healthy conditions or contribute to metabolic disorders in pathological conditions such as obesity, diabetes, cardiovascular diseases, or even cancer. These T cells play critical roles in immunometabolism, which refers to the intersection of immunity and metabolism. Numerous studies have examined the presence and changes of different T cell subsets, including helper T cells, regulatory T cells, cytotoxic T cells, and natural killer T cells, in adipose depots in health and diseases. In this review, we will discuss the adipose tissue niches that influence the patterns and functions of T cell subsets and in turn the impact of these T cells on cell- or body-based immunometabolism accounting for health and obesity.

## Introduction

Immunometabolism focuses on the interaction of immunity and metabolism, from metabolic patterns of immune cells to metabolic homeostasis or disorders dominated by immune cells. The former, as reviewed by Man et al. (1), is considered *cellular immunometabolism*, which includes the intracellular metabolism of different immune cells or immune cells in different conditions, such as macrophages or T cells during activation, polarization, proliferation, and differentiation. The latter is *tissue immunometabolism*, which explores the impacts of immune cells on tissue and systemic metabolism in various microenvironments (1–4). The immune system, which is influenced by the metabolic status of the body, in turn produces substantial impacts on local and systemic metabolic homeostasis or disorders. In recent years, the effects of immune responses on metabolic abnormalities such as obesity, diabetes, and fatty liver disease have drawn great interest from researchers, and emerging evidence has suggested that adipose tissue serves as an important inter-section linking immunity with metabolism (4, 5). Besides its traditional function in fat storage, adipose tissue is currently recognized as an endocrine organ (6–8). Importantly, accumulating data have shown that adipose tissue contains a large number of immune cells, including macrophages, eosinophils, innate lymphoid cells (ILCs), T cells, and B cells, which regulate immune homeostasis and inflammation, subsequently influencing metabolism of adipose tissue and the whole body (4, 5, 9–11). As the core component of adaptive immunity, T cells play indispensable roles in tissue immunometabolism. Here, we review the distinct profiles of T cell subsets in specific adipose tissue microenvironments and their influences on metabolic homeostasis or disorders.

## Adipose tissue provides a stage for the interplay between immunity and metabolism

There are two types of adipose tissue in mammals, white adipose tissue (WAT) for energy storage in the form of fat (triglyceride) and brown adipose tissue (BAT) for energy dissipation through thermogenesis. WAT is widely distributed throughout the body including subcutaneous adipose tissue (SAT) underneath the skin and intra-abdominal fat depots known as visceral adipose tissue (VAT) (12–14). Human SAT predominantly exists in the areas of abdomen, leg, and buttock, whereas VAT is mainly around omentum, mesenterium, and perirenal areas (12, 14). The initial link between WAT and immune function was demonstrated in several studies that revealed an association of obesity with WAT inflammation (15). Hotamisligil and colleagues demonstrated that the proinflammatory cytokine tumor necrosis factor (TNF)-α is elevated in VAT in obese animals compared with their lean counterparts and plays critical roles in obesity-induced insulin resistance (15, 16). In humans, the level of TNF-α is also increased in fat tissues from obese individuals and positively correlated with hyperinsulinemia (16). In addition, the proinflammatory cytokine interleukin (IL)-6 from adipose tissue also contributes to obesity-induced insulin resistance in both humans and mice (17, 18). Adipocytes are able to produce numerous inflammatory molecules, including TNF-α and IL-6, whereas macrophages appear to be the major source of these proinflammatory cytokines in adipose tissue *in vivo* as demonstrated in a mouse model (19). Indeed, macrophages were first reported to be increased and to polarize into classically activated M1-like phenotype in adipose tissues in obese humans and animals (6, 19–22). The chemokine monocyte chemoattractant protein-1 (MCP-1) is elevated and may contribute to the infiltration of macrophages in obese WAT and subsequently to obesity-induced insulin resistance (21, 23–25). Subsequently, T cells were found to be elevated in adipose tissue in obese mice and humans (26), and effector T cells, including CD4^+^ helper T (Th) cells and CD8^+^ cytotoxic T lymphocytes (CTLs), may serve as active players in obesity-associated WAT inflammation (27–30). In addition, several other immune cell populations or subsets mainly associated with type 2 immune response, such as type 2 innate lymphoid cells (ILC2), alternatively activated M2 macrophages, eosinophils, invariant natural killer T (iNKT) cells, and regulatory T or B cells, reside in adipose tissue under normal conditions but are reduced in obesity (31–35). These type 2 immune cells may be involved in maintenance of both immune and metabolic homeostasis under normal conditions. Energy excess or obesity can cause the disruption of this homeostasis and induce a new immune cell profile in adipose tissue that drives adipose tissue inflammation, insulin resistance, and related metabolic disorders.

## Various T cell subsets in different adipose tissue niches

Based on the composition of T-cell antigen receptors (TCR), T cells can be classified into two populations, αβT cells and γδT cells, both of which perform critical immune functions. While αβT cells serve in adaptive immunity, γδT cells act mainly in innate immunity. According to the cell surface markers, αβT cells can be further divided into two subsets: CD4^+^ T cells and CD8^+^ T cells. After activation by antigen stimulation, T cells can proliferate and differentiate into effector T cells. CD4^+^ T cells differentiate into effector Th cells and CD8^+^ T cells differentiate into CTLs, thus exerting distinct effects. An important regulatory subset among CD4^+^ T cells is regulatory T (Treg) cells, which have a specific molecular signature as CD4^+^ CD25^+^ Foxp3^+^. Treg cells inhibit the activation of T cells and the functions of effector T cells as well as B cells and NK cells, participating in the maintenance of tissue homeostasis and self-tolerance, or in the pathogenesis of some morbidities through negatively regulating immune responses (36).

The implication of T cells in obesity-induced inflammation was first indicated by the increased T cell accumulation in VAT in obese mice and humans as compared with their lean counterparts (26). The chemokine CCL5 (also known as regulated on activation, normal T cell expressed and secreted [RANTES]) is upregulated in VAT in obesity and may account for the recruitment of T cells into obese VAT (26, 37, 38). Importantly, T cells are increased early, likely preceding the infiltration of macrophages, in VAT in mice on high-fat diet (HFD), and play important roles in macrophage recruitment and VAT inflammation (30, 39, 40). While different effector T cell subsets are implicated in adipose tissue inflammation, regulatory T cell subsets are involved in healthy or normal adipose tissue homeostasis (31). Given the heterogeneity of T cells, we will discuss in this section the various patterns and functions of different T cell subtypes in adipose tissue niches.

## Treg cells serve to maintain adipose tissue homeostasis

The first finding regarding adipose-resident Treg cells was from Feuerer and colleagues, who reported an enrichment of CD4^+^ Foxp3^+^ Treg cells in VAT from lean mice (31). Besides the canonical gene signature such as Foxp3, CD25, glucocorticoid-induced tumor necrosis factor receptor (GITR), cytotoxic T lymphocyte antigen-4 (CTLA-4), and OX40, these Treg cells in VAT possess a phenotype different from those residing in lymphoid tissues, with distinct expression patterns of many Treg signature genes such as CD103 and G protein–coupled receptor−83 (31). Treg cells are markedly reduced in VAT of mice with diet-induced obesity. In addition, depleting Treg cells in lean mice induces the gene expression of inflammatory mediators (such as TNF-α, IL-6, and CCL5) and impairs the metabolic signal pathway in VAT, whereas expanding Treg cells in HFD-fed obese mice improves metabolic parameters, possibly through the regulation of adipose tissue inflammation, suggesting that Treg cells play crucial roles in the maintenance of immune and metabolic homeostasis of adipose tissue and may have beneficial effects on systemic metabolic abnormalities associated with obesity (31, 41).

**Figure 1:**
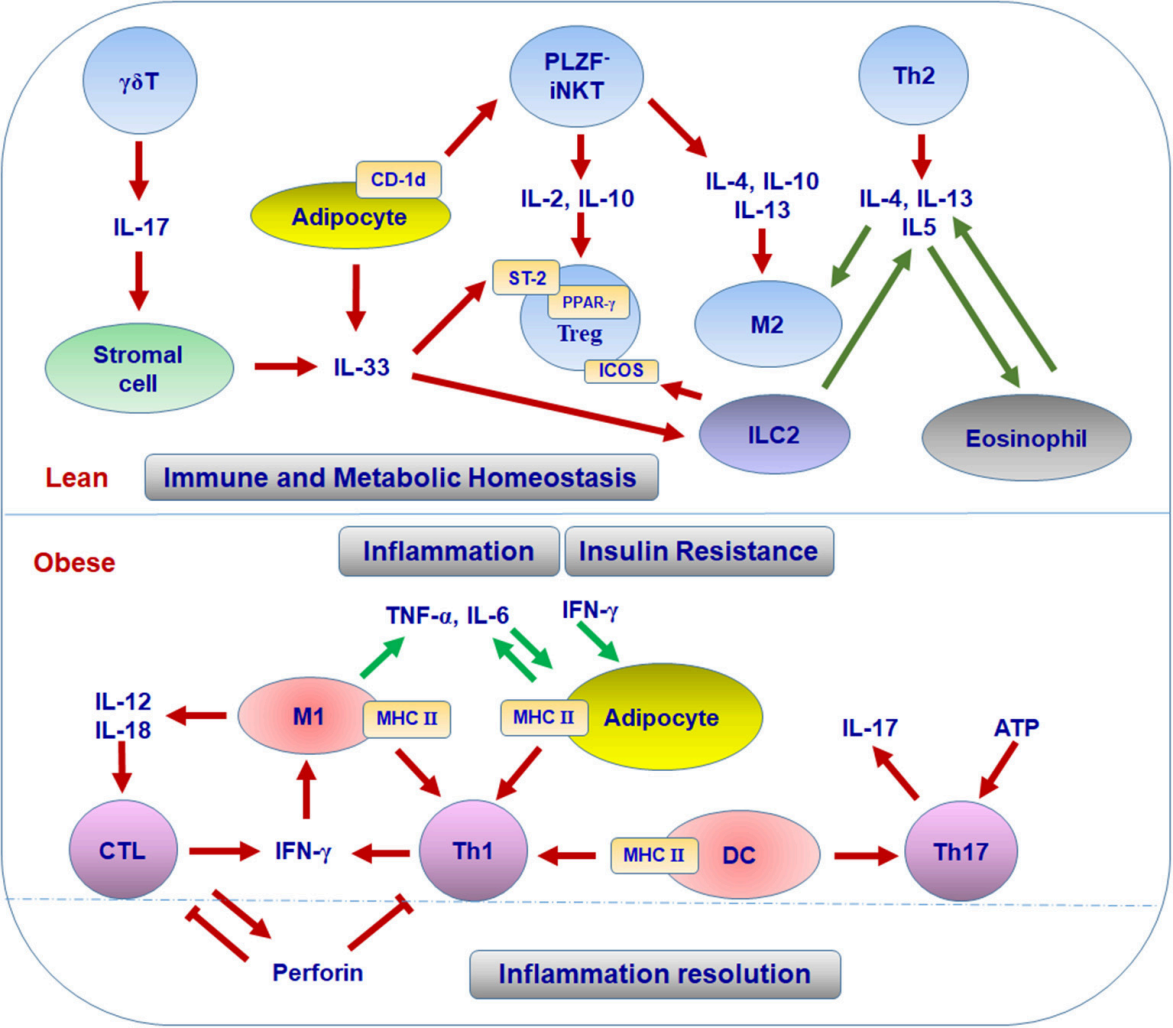
T cell subsets in different adipose niches in lean and obese conditions.

The mechanisms for the enrichment and function of Treg cells in lean VAT have not been fully defined. To date, several factors are considered to be critical for the maintenance of Treg cells in VAT. First, peroxisome proliferator-activated receptor-γ (PPAR-γ) expressed by Treg cells is necessary for the accumulation, phenotype, and function of VAT Treg cells in lean mice through collaborating with Foxp3 to induce a distinct Treg signature; while obesity induces the disappearance of this VAT Treg signature by phosphorylation of PPAR-γ at position Ser273 (42–44). These findings established a foundation for adipose Treg cells and opened a new area of research to elucidate the precise mechanisms by which PPAR-γ regulates VAT Treg signature. Second, the IL-33/suppression of tumorigenicity 2 (ST-2) axis plays an essential role in the amplification of Treg cells in VAT (45–47). IL-33 is a cytokine of the IL-1 family and can be produced by human adipocytes or mice stromal cells in VAT; ST-2, the receptor for IL-33, is highly expressed on VAT Treg cells in both humans and mice (45, 46, 48, 49). IL-33 can promote the development and proliferation of Treg cells and then restore their numbers in VAT, with attenuation in VAT inflammation and improvements in the metabolic parameters in obese mice (45–47). More recently, Kohlgruber and colleagues reported that adipose-resident γδT cells positive for the Broad-complex, Tramtrack, and Bric-à-brac/poxvirus and zinc finger (BTB-POZ) transcription factor PLZF produce IL-17, which induces IL-33 expression from adipose stromal cells, thereby contributing to age-dependent Treg cell accumulation in adipose tissue (49). The same research group also found that *i*NKT cells, a unique regulatory population residing in adipose tissue, help sustain the immune homeostasis of adipose tissue through regulating the number and function of Treg cells (50) (see discussion below). In addition, IL-33 can active ILC2, which promotes Treg cell accumulation in VAT through the ligation of ICOSL on ILC2 with ICOS on Treg cells. This process can be suppressed by interferon (IFN)-γ, which elicits VAT inflammation and metabolic disorders (51). Thus, it is acknowledged that the accumulation of Treg cells in VAT is a multifactorial process, which is further substantiated by a recent report showing the collaboration of TCR, Foxp3, and ST2 in this process (52). Some other factors such as leptin, IL-21, and autophagy-related atg7 have been reported to influence Treg cells in VAT and systemic insulin sensitivity (53–55). However, the direct link and underlying mechanisms remain to be established. Taken together, the available data indicate that various cells and molecules form specific adipose tissue niches that contribute to the pool and function of adipose Treg cells and maintain the homeostasis of systemic metabolism (Figure 1).

## Th cells mediate divergent immune and metabolic effects on adipose tissue through different subtypes

Based on the stimuli, Th cells can polarize into different phenotypes that express distinct cytokine profiles and exert different effector functions. Th1 cells produce IFN-γ, which promotes the polarization of classically activated M1 macrophages, and participate in elimination of intracellular microbes. Th2 cells produce IL-4, IL-5, and IL-13 to promote the polarization of alternatively activated M2 macrophages, help B cell responses, and participate in the immune responses against helminths. Th17 cells produce IL-17 to induce neutrophil inflammation and participate in the pathogenesis of several autoimmune diseases (36).

### Th1 subset

The first Th subset identified in adipose tissue was Th1 cells. The infiltration of CD4^+^ T cells was initially identified in human VAT and showed significant correlation with the body mass index (39). Furthermore, waist circumference correlated with IFN-γ mRNA in SAT from patients with type 2 diabetes, suggesting an association of adipose tissue Th1 cells with obesity (39). CD4^+^ T cells are also increased in VAT of obese mice compared with lean controls. Importantly, CD4^+^ T cells from obese VAT produce higher amounts of IFN-γ than those from lean VAT, indicating Th1 polarization in obese VAT (29, 40, 56–58). Furthermore, deficiency of T cells, including CD4^+^ T cells or IFN-γ significantly reduces adipose tissue inflammation and improves insulin sensitivity in obese mice, suggesting the substantial contribution of Th1 cells to adipose inflammation and metabolic dysfunctions associated with obesity (29, 59). Th1 cells and IFN-γ, the major Th1 and CTL cytokine, can directly interrupt insulin signaling, leading to insulin resistance in adipocytes and skeletal muscle myocytes, which may contribute to systemic insulin resistance in obesity (29, 58, 60). As to the mechanisms for Th1 polarization in obese VAT, obesity increases the levels of class II major histocompatibility complex (MHC II) and costimulatory molecules on VAT macrophages/dendritic cells (DCs) and also on adipocytes. These adipocytes and macrophages/DCs can function as antigen-presenting cells (APCs) to promote adipose tissue CD4^+^ T cell proliferation and production of IFN-γ, which further activate adipocytes and macrophages/DCs with elevated MHC II, thereby forming a positive loop to amplify Th1 cell-mediated inflammation in adipose tissue (Figure 1) (39, 40, 57, 59, 61, 62). Importantly, adipocyte- or macrophage-specific depletion of MHC II in obese mice reduces CD4^+^ T cell numbers (especially the effector/memory CD4^+^ T cells) and IFN-γ production in VAT and improves insulin sensitivity, indicating critical roles of MHC II on adipocytes or macrophages in adipose tissue Th1 polarization, which contributes to obesity-induced adipose tissue inflammation and insulin resistance (40, 56, 63).

On the other hand, several pivotal costimulatory receptors on T cells such as 4-1BB and CD28 are reported to be implicated in obesity-induced adipose tissue inflammation and metabolic disorders, as evidenced by the fact that 4-1BB or CD28 deficiency in mice improves related metabolic parameters and reduces T cell infiltration in VAT (64, 65). Considering the crucial roles of costimulatory signals 4-1BB−4-1BBL and CD28–B7 in T cell activation and proliferation and Th1 polarization (66–68), it is reasonable to deduce the potential contribution of 4-1BB and CD28 to Th1 polarization in obese VAT; however, future studies are needed for confirmation.

ICOS is another inducible costimulatory receptor that can be broadly expressed by various T cells and implicated in the expansion and function of Th2 and Treg cells, and even Th1, Th17, and NKT cells (51, 69–73). The expression patterns of ICOS on adipose tissue T cells and its potential complex functions in lean and obese states are intriguing and warrant investigation. Furthermore, as exosomes from macrophages/DCs can carry MHC II or costimulatory molecules that help the activation and proliferation of T cells (74–76), it is very likely that APCs residing in VAT secrete exosomes to promote activation and further Th1 polarization of CD4^+^ T cells, thereby driving adipose tissue inflammation and insulin resistance. This is a promising field of research that deserves further exploration and may help to delineate the mechanisms for Th1 polarization in obese VAT.

### Th17 subset

A few earlier studies reported the association of obesity with IL-17 or Th17 cells (77–79). However, these studies lacked conclusive evidence for the presence of Th17 cells in adipose tissue. First, besides Th17 cells, γδT cells are another important source of IL-17. Therefore, the role of IL-17 in obesity cannot be completely attributed to Th17 cells and needs to be further elaborated. Second, the increase of Th17 subset in lymphoid tissues, including spleen and lymphoid nodes, in obesity, or in concert with other medical conditions like experimental allergic encephalomyelitis or trinitrobenzene sulfonic acid colitis may be distinguished from the presence of Th17 subset in adipose tissue, and additional studies are needed to explain fully the influence of Th17 cells on immunometabolism (77–79). Nevertheless, Bertola and colleagues found that IL-17–producing T cells are dramatically increased in SAT from overweight or obese individuals compared with that from lean subjects and further demonstrated that DCs from obese subjects may drive the differentiation of Th17 cells, which produce high amounts of IL-17, indicating that Th17 cells may directly participate in adipose tissue inflammation and insulin resistance in obesity (80). In addition, several more studies confirmed the elevation of Th17 cells in adipose tissue, especially in VAT, in patients with adiposity (81–83). More recently, a study showed that ATP drives the Th17 responses via P2X7 receptor in human VAT, pointing to a possibility that adipose tissue niches facilitate the differentiation of Th17 cells (84). However, more studies are needed to verify the profile, functions, and underlying mechanisms of Th17 cells in adipose tissue and the direct contribution of Th17 cells to adipose tissue inflammation and insulin resistance.

In addition to Th17, γδT cells that reside in adipose tissue and are increased in obesity (85), also express IL-17. As mentioned above, adipose γδT cells can contribute to the regulation of age-dependent adipose tissue Treg homeostasis through IL-17–induced IL-33 production from adipose stromal cells (49). Thus, based on the increases of both Th17 and γδT cells in obese adipose tissue and the effect of IL-17 on IL-33 production, these two IL-17–producing cell subsets may also have the potential to initiate downregulation of the immune inflammatory reaction in adipose tissue during obesity, although this remains to be demonstrated.

### Th2 subset

Th2 cells produce type 2 cytokines, including IL-4, IL-5, and IL-13, which play important roles in macrophage polarization into M2 phenotypes. Several cell types, including eosinophils and ILC2, have been identified in lean adipose tissue to produce type 2 cytokines and may contribute to M2 polarization, inflammation resolution, and metabolic homeostasis in WAT under normal conditions (34, 35). However, data are limited on Th2 cells in adipose tissue. A study showed that the percentage of Th2 cells in human SAT and VAT negatively correlates with systemic inflammation and insulin resistance, indicating a protective role of Th2 cells in inflammation and metabolic dysfunctions (83). Another study revealed that after adoptive transfer into obese Rag1-null mice, CD4^+^ T cells gained a Th2 profile, indicated by the production of IL-4 and IL-13, which was associated with reversal of enhanced weight gain and insulin resistance in recipient obese Rag1-null mice. Consistently, transfer of CD4^+^ T cells deficient in signal transducer and activator of transcription 6 (STAT6), a transcription factor important for Th2 polarization, into obese Rag1-null mice resulted in the reduction in Th2 cells in VAT and the loss of protective effects on obesity-related metabolic parameters in recipient Rag1-null mice (86).

## CTLs perform bidirectional function in adipose tissue inflammation and homeostasis

Similar to CD4^+^ T cells, CD8^+^ T cells are significantly increased in adipose tissue in obesity in both humans and mice (28, 30, 59, 87, 88). Along with macrophages, CD8^+^ T cells participate in formation of crown-like structures (CLSs) surrounding dying/dead adipocytes in adipose tissue of mice (30). Increased infiltration as well as IL-12– and IL-18–mediated proliferation and activation may contribute to the increase and activation of CD8^+^ T cells in adipose tissue in obese mice (28). The increase in adipose tissue CD8^+^ T cells appears to precede and contributes to the accumulation of adipose tissue macrophages and metabolic dysfunctions in obesity. In support of this, depletion of CD8^+^ T cells in obese mice dramatically decreases numbers of M1 macrophages and CLSs in adipose tissue, accompanied by an improvement of insulin sensitivity, whereas adoptive transfer of CD8^+^ T cells into CD8-deficient mice fed with HFD increases numbers of adipose tissue macrophages and CLSs, with elevated levels of proinflammatory cytokines and aggravated insulin resistance (30). The increase of IFN-γ-expressing CD8^+^ T cells in VAT in obesity further substantiates the contribution of CD8^+^ T cells to macrophage activation through the action of IFN-γ (28, 30, 89, 90).

Besides their contribution to adipose tissue inflammation, effector CTLs may also function in restricting T cell expansion and activation in inflamed WAT through perforin. Perforin-dependent cytotoxicity is not only an important way to attack target cells, but also a critical regulator to limit abnormal T cell activation in a physiological context (91, 92). In mice fed HFD, depletion of perforin causes aggravated adiposity and insulin resistance, together with upregulation of IFN-γ-producing CD4^+^ and CD8^+^ T cells as well as M1 macrophages in VAT. Perforin-deficient CD8^+^ T cells from VAT show increased proliferation but impaired early apoptosis. Transfer of perforin-deficient CD8^+^ T cells into CD8-deficient mice exacerbates the metabolic parameters more than wild-type CD8^+^ T cells (93). These findings suggest that CTLs in fat tissue not only mediate adipose tissue inflammation in obesity, but may also contribute, at least partially, to the resolution of T cell–mediated inflammation through perforin-dependent cytotoxicity (Figure 1).

## NKT cells maintain the immune and metabolic homeostasis in adipose tissue

NKT cells are a unique subset of T cells that express both NK cell markers (such as NK1.1 or CD56) and T cell marker αβTCR. The main function of NKT cells is to recognize glycolipid antigen presented by MHC-class-I-like molecule CD1d. Based on the expression of an invariant TCRα chain (Vα14-Jα18 in mice, Vα24-Jα18 in humans), CD1d-dependent NKT cells can be classified into type I and type II NKT cells, both of which can produce IFN-γ, the Th1 cytokine, and IL-4, a Th2 cytokine, and participate in the regulation of innate and adaptive immunity. Type I NKT cells express the invariant TCRα chain in combination with certain TCRβ chains (Vβ8.2,7,2 in mice, Vβ11 in humans) and are also called iNKT cells, whereas type II NKT cells do not express this invariant TCRα chain (94–96).

Compared with other organs, adipose tissue in both humans and mice is enriched with iNKT cells under normal conditions, whereas obesity dramatically decreases iNKT cells in adipose tissue (97, 98). Accordingly, weight loss restores adipose tissue iNKT cells in murine models and peripheral iNKT cells in obese humans (97, 98). Huh and colleagues reported that the maintenance of iNKT cell numbers and activation in adipose tissue relies on their interaction with CD1d expressed on adipocytes. Adipocytes with high expression of CD1d under normal conditions function as APCs to present lipid antigens to maintain iNKT cells in adipose tissue and stimulate their activation, whereas obesity reduces CD1d expression in human and mouse adipose tissue, leading to the reduction of adipose tissue iNKT cells (99–101).

Depletion of iNKT cells (deficient in Jα18) or deficiency of CD1d in mice exacerbates HFD-induced weight gain, adipocyte hypertrophy, fatty liver, and insulin resistance as compared with wild type controls, whereas adoptive transfer of iNKT cells into obese Jα18-deficient mice or activating iNKT cells by lipid ligand α-galactocylceramidee (αGalCer) in obese wild-type mice reverses HFD-induced phenotypes, with reduced weight gain and adipocyte hypertrophy, and alleviated fatty liver and insulin resistance. These effects indicate a protective role of iNKT cells in HFD-induced weight gain and metabolic dysfunctions. Adipose tissue–resident iNKT cells express the transcription factor E4BP4, but not the BTB-POZ transcription factor PLZF. Under normal conditions, these adipose tissue iNKT cells produce high levels of IL-2 and type 2 cytokines such as IL-4, IL-10, and IL-13, but low level of IFN-γ, as compared to iNKT cells from the spleen. Indeed, the type 2 cytokines are downregulated in VAT of mice with CD1d deficiency and upregulated by αGalCe treatment, which is consistent with changes in the numbers of iNKT cells in VAT, suggesting that iNKT cells make substantial contributions to the levels of regulatory type 2 cytokines in VAT. These type 2 cytokines may inhibit the infiltration and activation of proinflammatory M1-like macrophages, but enhance polarization of M2 macrophages as well as expansion and suppressive function of Treg cells, thereby maintaining immune homeostasis and alleviating inflammation in adipose tissue (50, 98, 102). Adipocyte-specific deficiency of CD1d in obese mice attenuates the responses of iNKT cells to αGalCer, leading to reduced expression of IL-4 and IL-2 in iNKT cells, subsequent impairment of the anti-inflammatory responses mediated by M2 macrophages and Treg cells, and aggravation of adipose tissue inflammation and insulin resistance (99–101). All these findings support an important role of iNKT cells in maintaining adipose tissue homeostasis under normal conditions and in protecting against adipose tissue inflammation and metabolic dysfunctions associated with obesity, possibly through producing type 2 cytokines (Figure 1).

In addition, iNKT activation–mediated weight loss and improvement of insulin sensitivity in obese mice may also be attributable to β-oxidation–mediated energy expenditure and thermogenesis. Mechanistically, activation of iNKT with αGalCer treatment strongly induces the expression and production of FGF21 in both BAT and inguinal SAT, which drives the activation of BAT and browning of WAT to burn fat through β-oxidation. These findings point to another potential mechanism for the beneficial roles of iNKT cells in adipose tissue to maintain metabolic homeostasis through thermogenesis (103). However, a potential connection between iNKT effects on thermogenesis and inflammation remains to be clarified.

Taken together, these observations indicate that adipose tissue iNKT cells, as a unique regulatory immune cell subset, play important roles in both immune regulation and lipid metabolism to maintain the homeostasis of immunometabolism.

## Questions and perspectives

T cells reside in the network of adipose tissue, in which different types of cells interact with each other through the action of various cytokines, adipokines and membrane receptors. Beyond the information that is already known, additional components of the network may influence the profile and functions of T cells in adipose tissue. For examples, it remains an open question whether unknown or newly-discovered T cell subsets such as Th9 and Th22 cells exist and function in adipose tissue. The distinct signatures and regulatory mechanisms of well-recognized adipose T cell subsets, including CTL, Th1, and Th17 cells, need to be elaborated. Given the direct participation of iNKT cells in thermogenesis and of Treg cells in lipid uptake (42, 103), precise elucidation of metabolic functions of various T cells in physiopathological adipose tissue may provide new insight for the direct contribution of T cells to metabolism beyond immunity. Moreover, the interactions of T cells with other, non-immune cells in the adipose stromal vascular fraction, such as stem cells or endothelial cells, may also be crucial events that impact the profile and functions of T cells. It has been demonstrated that adipose-derived stem cells from lean mice regulate macrophage polarization, thereby reducing adipose tissue inflammation, whereas those from obese subjects induce Th17 cells and activate monocytes, thus promoting inflammation (104–106). Therefore, it is important to examine the effects of these cells on different adipose T cell populations, which may link immunity with metabolism in adipose tissue in a different manner. Finally, given the discussed roles of various types of T cells in obese adipose tissue, mainly observed in animal models and tissue culture, it is important to explore the feasibility of targeting these immune cells as new therapies for obesity-related metabolic disease in humans.

## Concluding remarks

**Figure 2:**
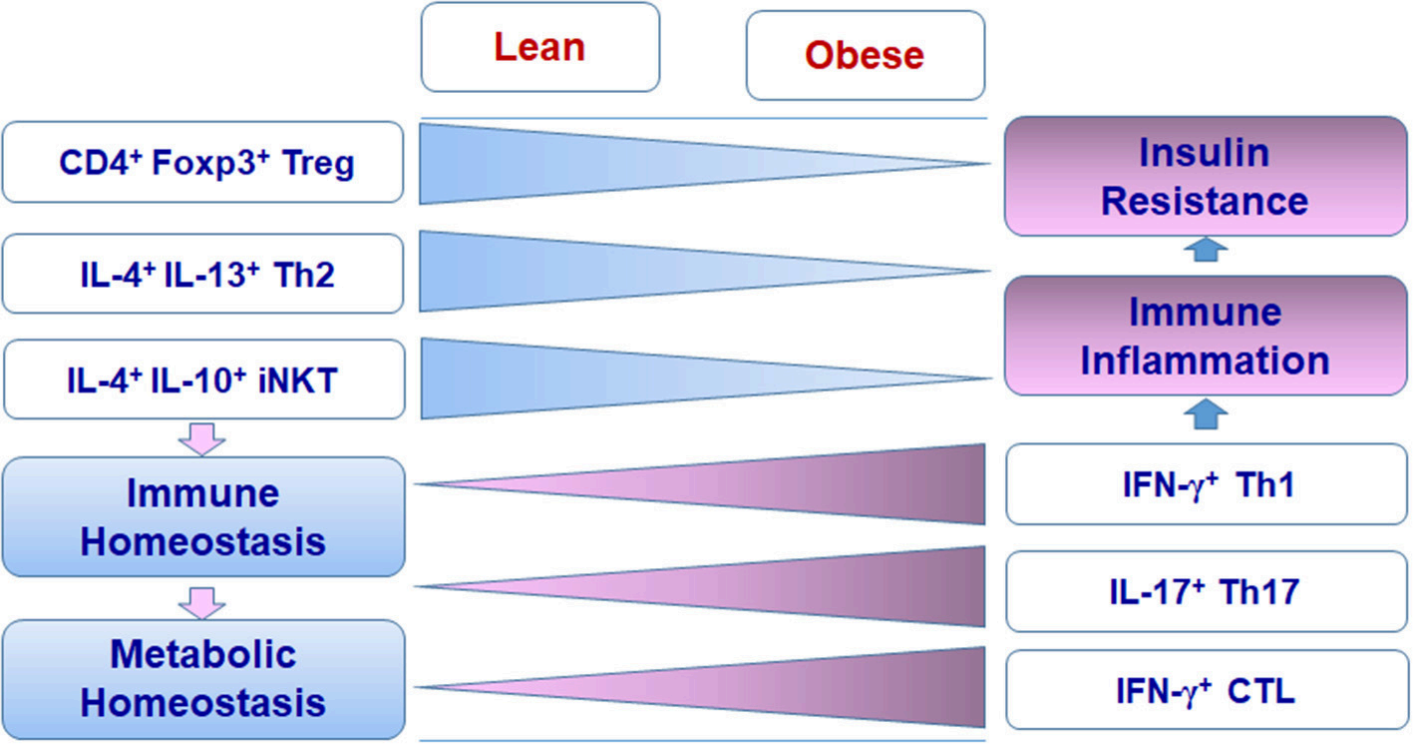
Changes of T cell subsets in adipose tissue in obesity.

Adipose tissue performs complex functions related to metabolism, immune responses, and endocrine effects. Besides adipocytes and preadipocytes, adipose tissue includes various immune cells that compose special adipose niches under different physiological or pathological conditions. T cells function as critical players in adipose tissue and influence the balance and functions of various populations of immune cells, exerting beneficial or detrimental effects on immunometabolism. In the healthy state, Treg cells, Th2 cells, and iNKT cells work with other regulatory immune cells such as M2 macrophages, ILC2, and eosinophils to maintain the immune and metabolic homeostasis of adipose tissue, providing a steady environment to retain normal systemic metabolism. When obesity develops, Th1 cells, Th17 cells, and CTLs accumulate in adipose tissue and, along with other proinflammatory immune cells such as M1 macrophages, disrupt the immune homeostasis, causing adipose tissue inflammation and systemic insulin resistance (Figures 1, 2). The diversity of T cell pools in adipose tissue, either as friend or foe, may result from the change of metabolism and in turn influence metabolism in various ways.

## Author contributions

All authors listed have made a substantial, direct and intellectual contribution to the work, and approved it for publication.

### Conflict of interest statement

The authors declare that the research was conducted in the absence of any commercial or financial relationships that could be construed as a potential conflict of interest.
